# Suppression and Rebound of Pallidal Beta Power: Observation Using a Chronic Sensing DBS Device

**DOI:** 10.3389/fnhum.2021.749567

**Published:** 2021-09-09

**Authors:** Jackson N. Cagle, Joshua K. Wong, Kara A. Johnson, Kelly D. Foote, Michael S. Okun, Coralie de Hemptinne

**Affiliations:** ^1^Department of Neurology, University of Florida, Gainesville, FL, United States; ^2^Norman Fixel Institute for Neurological Diseases, Gainesville, FL, United States; ^3^Department of Neurosurgery, Norman Fixel Institute for Neurological Diseases, University of Florida, Gainesville, FL, United States

**Keywords:** deep brain stimulation, Parkinson’s disease, local field potential, electrophysiology, beta power oscillations

## Abstract

Pallidal deep brain stimulation (DBS) is an increasingly used therapy for Parkinson’s disease (PD). Here, we study the effect of DBS on pallidal oscillatory activity as well as on symptom severity in an individual with PD implanted with a new pulse generator (Medtronic Percept system) which facilitates chronic recording of local field potentials (LFP) through implanted DBS lead. Pallidal LFPs were recorded while delivering stimulation in a monopolar configuration using stepwise increments (0.5 mA, every 20 s). At each stimulation amplitude, the power spectral density (PSD) was computed, and beta power (13–30 Hz) was calculated and correlated with the degree of bradykinesia. Pallidal beta power was reduced when therapeutic stimulation was delivered. Beta power correlated to the severity of bradykinesia. Worsening of parkinsonism when excessive stimulation was applied was associated with a rebound in the beta band power. These preliminary results suggest that pallidal beta power might be used as an objective marker of the disease state in PD. The use of brain sensing from implanted neural interfaces may in the future facilitate clinical programming. Detection of rebound could help to optimize benefits and minimize worsening from overstimulation.

## Introduction

Deep brain stimulation (DBS) is an invasive neurosurgical therapy which can be applied for select movement and neuropsychiatric disorders. The classical procedure consists of implanting electrodes in the brain and delivering continuously electrical stimulation through an implanted battery source referred to as an impulse generator. Although the subthalamic nucleus (STN) has been the most common brain region targeted, the globus pallidus internus (GPi) has been increasingly used especially in cases with dyskinesia, cognitive issues and a need for long-term medication adjustment flexibility (Okun et al., 2009; Follett et al., 2010).

One challenge in DBS treatment has been the complexity of choosing the optimal therapeutic settings. DBS therapy can be adjusted by changing the stimulation frequency, pulse width, and amplitude which is deleivered via a standard square wave pulse. There are thousands of possible DBS programming combinations making algorithms important to drive practical delivery of care in the outpatient setting (Kuncel and Grill, 2004; Anderson et al., 2018). The complex therapeutic options can lead to laborious programming sessions aimed at identification of the optimal stimulation settings. Several algorithms have been developed in effort to improve the therapeutic setting selection including techniques which employ a volume of tissue activation analysis (Frankemolle et al., 2010; Krack et al., 2019) along with local field potentials (LFP) (Hoang et al., 2017).

A number of studies have shown that STN beta power (13 – 30 Hz) is correlated with Parkinson’s disease (PD) symptom severity, particularly rigidity and bradykinesia (Weinberger et al., 2006; Ray et al., 2008), and that the pathological beta signal will be attenuated by effective therapeutic STN stimulation (Bronte-Stewart et al., 2009). Recent advancements in the DBS technology have provided broader access to neural activity near the electrode and access during actual stimulation (Goyal et al., 2021). This advance has the potential to facilitate translation of existing research findings into the clinical environment and to enable the development of more objective approaches for DBS programming. Using a chronic sensing-enabled neurostimulator recently approved for commercial use (Medtronic Percept), Feldmann and colleagues observed robust changes in the STN beta power during stepwise monopolar stimulation could be used as a method to select optimal therapeutic settings (Feldmann et al., 2021). Although GPi DBS is increasingly used for the treatment of PD, with a number of intraoperative pallidal physiology studies (Silberstein et al., 2003; Eisinger et al., 2020) and chronic recordings (Lu et al., 2020; Neumann et al., 2020) shown potential correlates of symptoms, chronic sensing GPi physiology remains largely unknown and its potential to assist in determining optimal therapeutic programming settings remains to be demonstrated.

We report a single PD participant receiving unilateral GPi DBS treatment implanted with a novel sensing-enabled neurostimulator. We recorded the neural signals in the GPi region during DBS treatment and we analyzed the relationship between the changes in neural signals and symptom improvement. We hypothesized that pallidal stimulation would reduce the beta power and correlate with symptom improvement and that this technique might be useful as an objective measure to guide future DBS programming.

## Methods

### Study Participant

This study was approved by the Institutional Review Board (IRB) of the University of Florida (IRB#202002433). A 57-year-old male with a 10 years history of PD was recruited and consented according to the Declaration of Helsinki. The written consent form was approved by the local ethical committee. The participant was diagnosed as the akinetic and rigid subtype of PD with minimal tremor symptoms. He was on 1100mg L-Dopa equivalent daily dose of PD medication prior to receiving his DBS therapy. The Unified Parkinson’s Disease Rating Scale (UPDRS) Part III motor score at baseline was 34 OFF medication and 21 ON medication. He was implanted with unilateral GPi DBS of left hemisphere.

### Surgical Procedure and Electrode Localization

A DBS electrode (Medtronic Model 3387) was implanted in the GPi, as previously described (Foote and Okun, 2005). The surgical planning was performed using a modified digital Schaltenbrand-Bailey deformable atlas overlay over an MRI FGATIR sequence for targeting (Sudhyadhom et al., 2009), and microelectrode recording was performed along the planned trajectory to verify the presence of GPi neurons along the trajectory (Mann et al., 2009). A month following the DBS electrode implantation, the impulse generator (Medtronic Percept), a chronic sensing-enabled neurostimulator, was implanted into the right subclavicular region. This DBS device is a commercially available non-rechargeable neurostimulator delivering constant current and was enabled for LFPs sensing.

The final position of the DBS electrode was verified using post-operative non-contrast CT head imaging which was fused with the pre-operative MRI FGATIR using a 3D affine transformation. The same modified digital Schaltenbrand-Bailey atlas used for DBS implantation surgery was used to verify the electrode position relative to the target region.

### Study Protocol

Electrophysiological recordings were collected during the initial monopolar review visit 1 week following neurostimulator implantation. The electrophysiological recordings were collected with a sampling rate of 250 Hz as limited by the neurostimulator. The participant arrived in the visit in OFF medication state (at least 12 h after the last medication intake). First, a 2-min baseline LFP recording off stimulation was performed to determine the baseline characteristics of the three non-adjacent bipolar recording contact pairs (0–2, 1–3, and 0–3). Second, the clinical programmer examined the effect of stimulation delivered at each contact sequentially from the most ventral to the most dorsal contact by slowly increasing the stimulation amplitude while pulse width and frequency were kept constant at 90 μS and 130 Hz until the participant reported persistent stimulation induced side effects. During stimulation testing using contact 1 and 2, the LFPs were recorded continuously from contact 0–2 and 1–3, respectively. The stimulation amplitude was increased by 0.5 mA increments starting at 0 mA up to the side effects threshold, with a minimum of a 20 s waiting period between each amplitude change. In addition to the LFP, spectral power at the pre-defined frequency was simultaneously recorded, with a 1.26 ms sensing blanking and was averaged over 3000 ms. The power band was defined as the maximum beta peak at rest (17.57 ± 2 Hz for contact 0–2 and 23.44 ± 2 Hz for contact 1–3) by visual inspection on the Medtronic programming tablet. The defined spectral band power was collected by Percept PC at a rate of 2 Hz (500 ms per data point) with 3000 ms averaging window. After the waiting period, the clinician performed an assessment of contralateral upper limb rigidity and bradykinesia using the UPDRS item 22 and 23, respectively, to evaluate the therapeutic benefit of each stimulation amplitude.

### Data Analysis

The LFPs collected were exported to a standard JSON format file that was parsed and imported to Python 3. The raw time-domain LFP recordings were filtered between 1 and 100 Hz to remove drift and stimulation artifacts, then transformed to time-frequency plot using standard spectrogram with 1-s Hamming window and 0.5-s overlap. To quantify the stimulation-induced power changes, 15 s of data free of artifact, excluding 2 s of data immediately before and after stimulation changes were used to avoid stimulation transition artifacts; these were selected at each stimulation amplitude and the power spectral density was computed using Welch’s periodogram method with 1-s hamming window and 0.5-s overlap. The beta power which was simultaneously streamed was also averaged at each stimulation amplitude and was correlated with the symptom severity score from UPDRS motor assessment by applying a Spearman’s correlation.

## Results

### Baseline Characteristics and Electrode Localization

**FIGURE 1:**
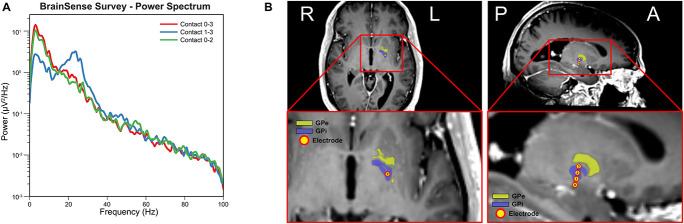
Electrode localization and baseline electrophysiology. **(A)** Power spectral density for each sensing-enabled contact pair showing a beta peak at 23.44 Hz for sensing contact pair 1–3 (data recorded during off stimulation). **(B)** Electrode contacts were identified in the fused post-operative CT image and pre-operative T1 MRI image. Electrode trajectory was close to a vertical trajectory (AC-PC angle 74-degree and central line angle 5-degree). Electrode contact 0 was outside of the GPi border while contact 1 to 3 were inside of GPi border. (Yellow atlas: GPe; Blue atlas: GPi; and Yellow dots: electrode contacts).

Figure 1A shows the power spectral density computed from the three sensing-enabled stimulation contact pairs (0–2, 1–3, and 0–3). A large peak in the beta band (13 – 30 Hz) was observed in bipolar sensing contact pairs 1–3, with the maximum power occurring at 23.44 Hz. Contact pairs 0–2 and 0–3 had minimal activity in the beta band and were instead characterized by stronger low frequency oscillations (1 – 10 Hz). These results suggested that contact 1 was likely localized closer to the beta source. Figure 1B shows the final electrode positions relative to the GPi. The most ventral contact (contact 0) was inferior to the GPi border, contacts 1 and 2 were within the GPi, and the most dorsal contact (contact 3) was located on the border of GPi and GPe.

### Beta Power Reduction With Pallidal Stimulation

**FIGURE 2:**
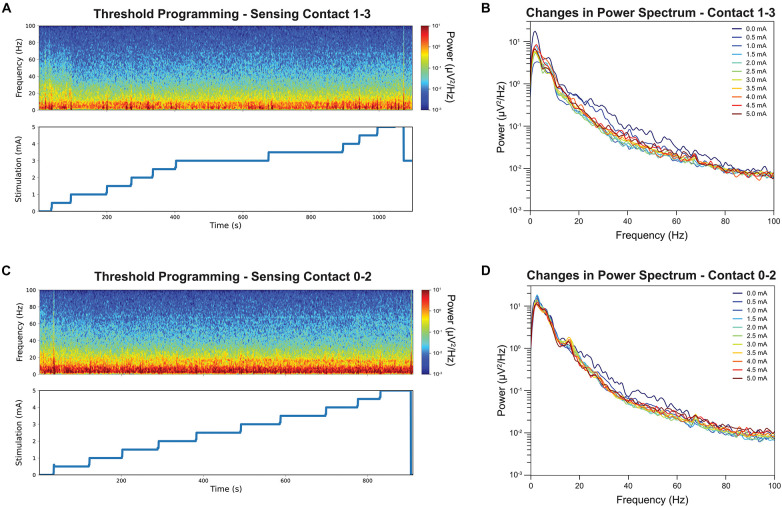
Time-frequency analysis during stimulation threshold testing and their corresponding stimulation amplitude is displayed. Note the stimulation amplitudes are up to but not exceedings the side-effect threshold for stimulation contact 2 **(A)**. The color denotes the absolute power at each time point, with the red indicating strongest power and the blue lowest. Sensing contact 1–3 revealed a reduction of power in the beta band in spectrogram when the stimulation amplitude was increased. The average power spectrum at each stimulation amplitude for stimulation contact 2 **(B)**. Increased stimulation amplitude was associated with a decrease in beta band power. The same time-frequency analysis was repeated for contact 1 as comparison **(C)**. Increased stimulation amplitude was associate with a smaller decrease in beta band power **(D)**.

Figure 2A shows the time-frequency plots of LFPs recorded from contact pair 1–3 while delivering stimulation from contact 2. The stimulation was slowly increased by a 0.5 mA increment from 0 mA to 5 mA, and hand tingling and mouth pulling was induced by the procedure at higher thresholds. The incremental stimulation amplitude is shown on the lower panel. Note that the stimulation changes were associated with transient artifacts. To quantify the effect of stimulation, the PSDs were computed at each stimulation amplitude, excluding a 2 s period at the stimulation transitions, and this is shown on Figure 2B. A reduction in beta and low gamma power with increased GPi stimulation was observed and we show this in both figures. Interestingly, a slight rebound in beta power was observed when stimulation amplitude was closer to the side-effect threshold.

Figure 2C shows the time-frequency plots of LFPs recorded from contact pair 0–2 while delivering stimulation from contact 1. Similar stepwise increment was performed at contact 1 and the stimulation PSDs were shown in Figure 2D. A narrow beta peak was observed at 17 Hz, but this peak was not correlated with symptom improvement. A reduction in broader high beta power was also found when stimulating from contact 1 and recording LFPs from contact 0–2 (Figure 2D) but not as significant as contact 2.

### Relationship of Beta Power and Symptom Severity

**FIGURE 3:**
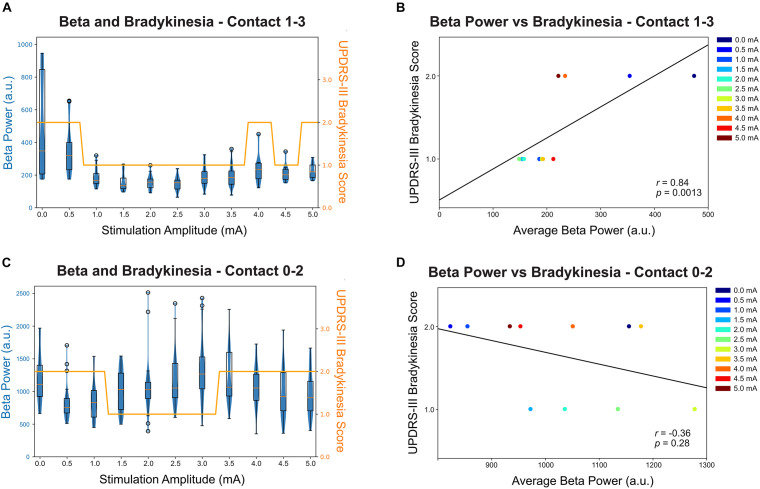
Relationship between beta power (recorded from contact 1–3 when stimulating at contact 2) and bradykinesia score (UPDRS Part 3 subitem 23). **(A)** The beta peak power (23.44 Hz) for contact 1–3 was averaged over each stimulation amplitude and shown in a box-violin plot with whisker at 150% of the interquartile distance and outliers were marked as circles. Each datapoint indicates a 500 ms power value collected by Medtronic Percept neurostimulator at the specified frequency band. The bradykinesia scores were plotted in the same scale for comparison. **(B)** The correlation between average beta power and bradykinesia score at each stimulation amplitude for contact 1–3. The beta power was statistically significantly correlated with bradykinesia scores, with increased beta power correlated with worse bradykinesia (*p* = 0.0013). **(C)** The beta peak power (17.57 Hz) for contact 0–2 was averaged over each stimulation amplitude and shown in a box-violin plot with whisker at 150% of the interquartile distance and outliers were marked as circle. **(D)** The correlation between average beta power and bradykinesia score at each stimulation amplitude for contact 0–2.

Of the two primary symptom measurements, only bradykinesia shows improvement with stimulation while rigidity remain at a score of 1 throughout the full therapy range; therefore, rigidity was not reported in the correlation analysis. Figures 3A,C shows both the distribution and the median of the average beta power (17.57 ± 2 Hz for contact 0–2 and 23.44 ± 2 Hz for contact 1–3) at each stimulation amplitude and this is displayed as a box-violin plot for LFP recorded from contacts 0–2 while stimulating on contact 1 and contacts 1–3 while stimulating on contact 2. Contact 2 best controlled the participant’s symptoms. Increased stimulation amplitude resulted in a reduction in beta power, with a considerable change at 1 mA at contact 2. Interestingly, the participant experienced a worsening of bradykinesia with a stimulation amplitude above 4.0 mA and the worsening was associated with increased beta power. The bradykinesia scores across stimulation amplitudes (Figure 3A) were significantly correlated with the average beta power values (Figure 3B) and analysis with a Spearman’s correlation coefficient revealed 0.84 (*p* = 0.0013). Contact 1 LFP does not show correlation with symptom improvement (Figure 3D, *p* = 0.28) and contact 1 therapy has a narrower therapeutic window than contact 2 (Figure 3C).

## Discussion

In this study, a novel sensing-enabled DBS device was applied to an individual with PD implanted with GPi DBS. LFPs were recorded while delivering stimulation, at contact 1 and 2 sequentially, and by slowly increasing stimulation amplitude until side effects were encountered. The bradykinesia severity and rigidity were recorded at each stimulation amplitude and was correlated with the LFP feature.

Real-time neuronal recordings during threshold testing revealed a beta desynchronization when electrical stimulation was delivered to the target region. The beta desynchronization was stronger for the sensing contact pair 1–3 than for contact pair 0–2. This matched the baseline characteristics which also revealed a stronger beta peak when sensing at contact pair 1–3. This result matched with the imaging which revealed that the electrode contacts were located within the GPi. The beta power was strongly correlated with bradykinesia improvement when we measured in the acute clinic setting. The improvement was consistent with previous intraoperative (Wang et al., 2018; Piña-Fuentes et al., 2019; Eisinger et al., 2020) and externalized lead studies (Burgess et al., 2010). However, rigidity score was not changed by the stimulation in either therapy contact pairs. Our results suggest that GPi beta power correlates with bradykinesia severity and might be used as an objective marker for selecting the optimal stimulation amplitude for treatment of bradykinesia. This procedure could be practical and useful for clinic-based settings.

Interestingly, the beta power rebounded when the stimulation amplitude was increased above 3 mA, and this rebound was associated with an increase in bradykinesia severity. Worsening of motor symptoms at higher stimulation amplitudes has been observed in previous clinical but not physiological studies (Baizabal-Carvallo and Jankovic, 2016; Hu et al., 2018), but the nature of these phenomena has remained unknown. The causal relationship as to whether the worsening of symptoms with overstimulation was induced by the beta increase or alternatively by induced side effects cannot be determined from our small dataset. However, the rebound of the beta power can provide useful information regarding the therapeutic window for DBS programming in addition to finding to therapy level that provide maximum benefits.

A limitation with the current protocol is the selection of peak beta frequency for tracking. In our study, contact pair 0–2 has a peak frequency at 17.57 Hz at baseline but this frequency is in fact uncorrelated to symptom improvement (Figure 3D). Although post-processing shows slight reduction of broader high beta power (Figure 2D), none of the frequency band were statistically correlating with symptom improvement nor capturing the rebound effect. Another limitation is the sequence of stimulation stepwise increment used in the protocol. The suppression and rebound of beta might be an effect of cumulative stimulation and using a protocol that randomize therapy amplitude during recording can account for the sequential relationship and reflect the true physiological behavioral of excessive stimulation. Thirdly, the clinician-rated bradykinesia scores and rigidity scores may not fully capture the minor changes in symptom severity. In the current study, rigidity severity has not changed with any level of stimulations while the bradykinesia severity only altered by one point. Objective measures such as sensor-based measurement may be able to capture the miniature changes and offer better correlation with neural signals.

This study provides preliminary evidence for the feasibility of using real-time neuronal recordings to choose optimal DBS programming settings. Future studies focusing on more long-term evaluation of biomarkers across a larger sample size will likely guide which individuals with PD GPi DBS may benefit from this technique and possibly which may possibly benefit from closed-loop stimulation.

## Data Availability Statement

The raw data supporting the conclusions of this article will be made available by the authors upon reasonable request.

## Ethics Statement

The studies involving human participants were reviewed and approved by University of Florida Institutional Review Boards. The patients/participants provided their written informed consent to participate in this study. Written informed consent was obtained from the individual(s) for the publication of any potentially identifiable images or data included in this article.

## Author Contributions

JC: data collection, data analysis, and drafting of article. JW: data collection and reviewing of article. KJ and MO: reviewing of article. KF: surgery and reviewing of article. CH: reviewing of article and concept development. All authors contributed to the article and approved the submitted version.

## Conflict of Interest

The authors declare that the research was conducted in the absence of any commercial or financial relationships that could be construed as a potential conflict of interest.

## Publisher’s Note

All claims expressed in this article are solely those of the authors and do not necessarily represent those of their affiliated organizations, or those of the publisher, the editors and the reviewers. Any product that may be evaluated in this article, or claim that may be made by its manufacturer, is not guaranteed or endorsed by the publisher.
